# Supply-side contribution to the lack of PBF impact on unmet need for family planning in Burkina Faso

**DOI:** 10.1093/eurpub/ckac131.431

**Published:** 2022-10-25

**Authors:** C Hertler, J Lohmann, JL Koulidiati, PJ Robyn, SMA Somda, M De Allegri, S Brenner

**Affiliations:** 1 Heidelberg Institute of Global Health, Heidelberg University, Heidelberg, Germany; 2 Department of Global Health and Development, LSHTM, London, UK; 3 The World Bank, Washington, DC, USA; 4 Centre MURAZ, Bobo-Dioulasso, Burkina Faso; 5 UFR/ST, Université Nazi Boni, Bobo-Dioulasso, Burkina Faso

## Abstract

**Background:**

In 2020, about one in four women in Burkina Faso faced an unmet need for family planning (FP). Between 2013 and 2017, Burkina Faso implemented a performance-based financing (PBF) program to improve primary health care service provision (including FP) at rural health centers. Our prior work revealed that PBF did not lead to a reduction in unmet need for FP, in spite of FP being an explicitly targeted service. Our current study assesses supply-side factors that have likely contributed towards this lack of effect at population level, by examining changes in facility-based indicators relevant to the provision of FP induced by PBF.

**Methods:**

We used facility-based survey data from 406 PBF and 117 control facilities collected before and after the PBF implementation. To compare changes in FP service provision, we examined changes in a number of relevant indicators including: a. the types of FP methods offered by facilities; b. trainings received by different FP providers; and c. available stocks of modern contraceptives. We relied on a difference-in-differences (DID) regression model to estimate the impact of PBF on these indicators.

**Results:**

We observed a significant positive impact on the number of staff qualified to provide injectables, implants and IUDs (effect size 0.47, p 0.003) as well as the number of facilities offering IUDs (effect size 0.28, p 0.016) and a significant reduction in the number of facilities experiencing stock-outs of female condoms (effect size -0.09, p 0.007) and implants (effect size -0.03, p 0.042).

**Conclusions:**

Given the significant positive impacts on the number of qualified staff, facilities providing IUDs and a reduction in stock-outs of female condoms and implants attributable to the PBF intervention without showing signs of negative effects on the indicators measured supply-side factors might not have been the main reason for the lack of effect of the PBF program on unmet need for FP.

**Key messages:**

• Supply-side factors might not have been the main reason for the lack of effect of the PBF program on unmet need for FP.

• Further research is needed to explore other potential underlying reasons.

